# Monoclonal Antibodies Specific to the Extracellular Domain of Histidine Kinase YycG of *Staphylococcus epidermidis* Inhibit Biofilm Formation

**DOI:** 10.3389/fmicb.2020.01839

**Published:** 2020-08-07

**Authors:** Zhihui Lyu, Yongpeng Shang, Xiaofei Wang, Yang Wu, Jinxin Zheng, Huayong Liu, Ting Gong, Lina Ye, Di Qu

**Affiliations:** ^1^Key Laboratory of Medical Molecular Virology of MOE and MOH, Department of Medical Microbiology and Parasitology, School of Basic Medical Sciences, Fudan University, Shanghai, China; ^2^Department of Infectious Diseases and Shenzhen Key Lab for Endogenous Infection, Shenzhen Nanshan Hospital, Shenzhen University, Shenzhen, China

**Keywords:** *Staphylococcus epidermidis*, biofilm, YycG histidine kinase, two-component system, epitope mapping

## Abstract

*Staphylococcus epidermidis* is frequently associated with biofilm-related infections. Biofilms drastically reduce the efficacy of conventional antibiotics and the host immune system. In *S. epidermidis* biofilm formation, a major role is played by the YycG/YycF two-component system, and previous findings have indicated that inhibitors targeting the cytoplasmic HATPase_c domain of YycG kinase in *S. epidermidis* exhibit bactericidal and biofilm-killing activities. Therefore, we hypothesized that monoclonal antibodies (mAbs) against YycG extracellular (YycG_ex_) domain would block the signal transduction and influence the biofilm formation of *S. epidermidis*. In this study, we screened out two YycG_ex_-specific mAbs showing the highest affinity for the target, mAbs 2F3 and 1H1. These mAbs inhibited *S. epidermidis* biofilm formation in a dose-dependent manner, and at a concentration of 160 μg/mL, mAbs 2F3 and 1H1 caused 78.3 and 93.1% biofilm reduction, respectively, relative to normal mouse IgG control. When co-cultivated with YycG_ex_ mAbs, *S. epidermidis* cells showed diminished initial-adherence capacity, and the antibody treatment further led to a marked decrease in the synthesis of polysaccharide intercellular adhesin and in the transcriptional level of genes encoding proteins involved in biofilm formation. Lastly, we determined that the epitopes recognized by the two YycG_ex_ mAbs are located within aa 59–70 of the YycG_ex_ domain. It indicates that the YycG_ex_ domain may be a potential candidate as a vaccine for the prevention of *S. epidermidis* biofilm infections.

## Introduction

*Staphylococcus epidermidis* is part of the normal bacterial flora colonizing the skin and mucous membranes of the human body (Yao et al., 2006; Fey and Olson, 2010). The main pathogenicity associated with *S. epidermidis* is the formation of thick, multilayered, highly structured biofilms on artificial surfaces (Vuong and Otto, 2002; McCann et al., 2008). Biofilm formation is a highly complex process that involves multiple regulatory factors, such as two-component systems (TCSs) (Sauer, 2003; Mikkelsen et al., 2011; Rasamiravaka et al., 2015; Wolska et al., 2016). The YycG/YycF TCS is conserved in low-G + C gram-positive bacteria (Fabret and Hoch, 1998; Fukuchi et al., 2000), and the TCS regulates bacterial growth, cell-wall metabolism, cell division, virulence-factor expression, and biofilm formation (Ng et al., 2005; Senadheera et al., 2005; Bisicchia et al., 2007; Fukushima et al., 2008). Because YycG/YycF TCS plays an essential role in bacterial biological functions and because no YycG/YycF homologs or structurally similar proteins are expressed in humans, the YycG/YycF TCS represents a potential antibacterial target (Winkler and Hoch, 2008; Gotoh et al., 2010).

The YycG histidine kinase of *S. epidermidis* prototypically contains two transmembrane sections, an extracytoplasmic loop (YycG_ex_ domain), cytoplasmic HAMP and Per-Arnt-Sim (PAS) domains, and a catalytic core of HisKA and HATPase_c domains (Ng and Winkler, 2004; Dubrac et al., 2008). Our previous work demonstrated that inhibitors targeting the HATPase_c domain exhibit not only bactericidal activity but also potent activity against staphylococcal biofilms (Qin et al., 2006; Huang et al., 2012; Liu et al., 2014; Lv et al., 2017). Thus, we hypothesized that monoclonal antibodies (mAbs) against the YycG_ex_ domain would block the signal transduction and thereby influence the phenotypic properties of *S. epidermidis* RP62A. Analysis of the YycG sequence revealed that the YycG_ex_ domain contains aa 142–154 flanked by two transmembrane domains that adopt a PAS or PhoQ-DcuS-CitA fold (Santelli et al., 2007; Chang et al., 2010; Shah et al., 2013). The folding motif is regarded as a sensor that receives environmental signals and modulates YycG kinase activity (Ng and Winkler, 2004; Szurmant et al., 2008). Accordingly, we generated mAbs against the YycG_ex_ domain and investigated their effects on bacterial functions.

Out of nine YycG_ex_-specific mAbs (2A9, 2B3, 2B4, 2F1, 2F3, 2E3, 2G9, 1H1, and 1H3) generated using hybridoma technology, two YycG_ex_ mAbs exhibiting the highest affinity for YycG_ex_ protein, mAbs 2F3 and 1H1, were selected for further experiments. Here, we investigated the anti-biofilm activities of these YycG_ex_ mAbs and identified the epitope recognized by the antibodies. Our results showed that YycG_ex_ mAbs inhibit *S. epidermidis* biofilm formation and that their target epitope is located within aa 59–70 of YycG_ex_ domain. We report, for the first time, that mAbs against the extracellular domain of a TCS can affect biofilm formation. Our findings indicate that the use of YycG_ex_ mAbs represents a potential treatment strategy against biofilm infection and, further, that the epitope recognized by YycG_ex_ mAbs could serve as a vaccine candidate for preventing *S. epidermidis* biofilm infections.

## Materials and Methods

### Bacterial Strains, Culture Media, and Antibiotics

*Staphylococcus epidermidis* RP62A was obtained from American Type Culture Collection (ATCC 35984), and *S. epidermidis* clinical isolates were collected from the Department of Laboratory Medicine, Affiliated Gulou Hospital, Medical College of Nanjing University. Staphylococcal strains were cultured in tryptic soy broth (TSB; Oxoid Ltd., Basingstoke, United Kingdom), and *Escherichia coli* strains DH5α and BL21(DE3) were cultured in Luria-Bertani (LB) broth (1% tryptone, 0.5% yeast extract, 1% NaCl). Kanamycin sulfate was obtained from Sigma Aldrich (St. Louis, MO, United States).

### Generation of mAbs Against the YycG_ex_ Domain

As noted in the introduction section, the *S. epidermidis* essential histidine kinase YycG contains these domains: two transmembrane sections, an extracytoplasmic domain, cytoplasmic HAMP and PAS domains, and the catalytic core of HisKA and HATPase_c domains. Our previous work showed that inhibitors targeting the HATPase_c domain, developed using a structure-based virtual-screening approach, exhibit both bactericidal activity and potent anti-biofilm activity in the case of staphylococci. Because the extracytoplasmic domain of the protein (YycG_ex_ domain) is considered to play roles in sensing external stimuli, we hypothesized that mAbs targeting the YycG_ex_ domain might block signal transduction by binding to this domain and thus influence bacterial biofilm formation. A 453-bp *Nco*I/*Xho*I DNA fragment encoding the YycG_ex_ domain (aa 34–184 of YycG) was amplified from the chromosomal DNA of *S. epidermidis* RP62A (GenBank accession number NC_002976) by using the primers *yycG*_ex_-F and *yycG*_ex_-R (Table 1). The *yycG*_ex_ DNA fragment was ligated into the pET28a (+) expression vector, and the resulting plasmid was transformed into *E. coli* DH5α competent cells. The YycG_ex_ protein was expressed in *E. coli* BL21(DE3) cells and induced (for 14 h at 25°C) by adding isopropyl β-D-1-thiogalactopyranoside (IPTG; Thermo Fisher Scientific, Waltham, MA, United States) to a final concentration of 0.8 mM. The bacterial cells were lysed by sonication and centrifuged, and the supernatant was used for protein purification by using Ni-NTA agarose (Qiagen, Los Angeles, CA, United States) according to the manufacturer’s instructions. Purified YycG_ex_ protein was dialyzed against Tris buffer-15% glycerol and then sterilized by passage through a 0.22-μm-pore-size filter (Millipore, Bedford, MA, United States). The recombinant YycG_ex_ protein was used for immunizing BALB/c mice, and YycG_ex_-specific mAbs were generated using the hybridoma technology by Abmart Inc. (Shanghai, China)^1^.

**TABLE 1:** Primers used in this study^a^.

Primers	Sequences (5′-3′)
**Primers used for expression of YycG_ex_ protein**	
^b^*yycG*_ex_-F	CATGCCATGG TCACGAATAGTTTAGAAAAGGAATT
^b^*yycG*_ex_-R	CCGCTCGAG CTGATTAATGTTGTTCAGCTGATT
**Primers used for qRT-PCR**	
*gyrB-*F	AGAAGAGGAAGTTAGAGAAGA
*gyrB-*R	GCATATCCACTGTTATATTGAAG
*icaA-*F	ATCAAGCGAAGTCAATCTC
*icaA-*R	CAGCAATATCCTCAGTAATCA
*icaD-*F	CCAGACAGAGGCAATATCC
*icaD-*R	CAATCATCACAACGATACAATACA
*icaB-*F	CATATTCCAGCAACAGGTT
*icaB-*R	GCAACAGATTGAGACGAAT
*icaC-*F	AGGCGTCGGAATGATGTTA
*icaC-*R	AGTTAGGCTGGTATTGGTCAA
*aap*-F	AACAACAACAACACCAACT
*aap*-R	GCCACCATAATGAACGATT
*bhp-*F	GTCTACTTGGCTCTTATGC
*bhp-*R	AGGTGTCTTCAGTTGTAATAC
*fbe-*F	AAGCACATTACCGTTGAT
*fbe-*R	CCTACTAATATCGTATCTCCAT
*sarA-*F	GTAATGAACACGATGAAAGAACT
*sarA-*R	GCTTCTGTGATACGGTTGT
*altE-*F	AACTGTGTCTGCTAATCG
*altE-*R	TTACCGCTATTGTAGTCTTC
*yycG-*F	ATAGTGATTCCTTCTTGTTAG
*yycG-*R	GACTTGTTGTTGTTCTGT
*yycF-*F	GATGGTATGGAAGTATGT
*yycF-*R	CTGGTTGTGAATAATGAC
**Primers used for truncation of the SUMO-tagged YycG_ex_**	
TF_34__–__184_-F	ACGAATAGTTTAGAAAAGGAATTAC
TF_34__–__184_-R	CTACTGATTAATGTTGTTCAGCTGATT
TF_34__–__83_-F	ACGAATAGTTTAGAAAAGGAATTAC
TF_34__–__83_-R	CTAATTCAAAAGGTCTTGGATATCCTT
TF_84__–__133_-F	GAATATGCGAATCGTCAAGAAATAG
TF_84__–__133_-R	CTACGTTTGCCCTAAGGAGAGCGCC
TF_134__–__184_-F	AATGATCATATGGTTCTTAAGGATT
TF_134__–__184_-R	CTACTGATTAATGTTGTTCAGCTGATTG
TF_34__–__58_-F	ACGAATAGTTTAGAAAAGGAATTAC
TF_34__–__58_-R	CTAGACGTCTAATTGCTTCGCATATTG
TF_47__–__71_-F	AAGAACATAACACAATATGCGAAGC
TF_47__–__71_-R	CTAGACTGAACCTTTATCTTTATCCTTA
TF_59__–__83_-F	AATATTGAAAAGGTTTATAAGG
TF_59__–__83_-R	CTAATTCAAAAGGTCTTGGATATCC
TF_47__–__58_-F	AAGAACATAACACAATAT
TF_47__–__58_-R	CTAGACGTCTAATTGCTTC
TF_59__–__70_-F	AATATTGAAAAGGTTTAT
TF_59__–__70_-R	CTAACCTTTATCTTTATCC
TF_71__–__83_-F	TCAGTCAACGCTCAAAAG
TF_71__–__83_-R	CTAATTCAAAAGGTCTTGGAT
**Primers used for site-directed mutagenesis of the SUMO-tagged T_34__–__83_**	
K47V/N48V/I49V-F	GCTGCTGCTACACAATATGCGAAGCAA
K47V/N48V/I49V-R	CTTGAAGTTATCGAGTAATTCCTTTTC
T50V/Q51V/Y52V-F	GCTGCTGCTGCGAAGCAATTAGACGTC
T50V/Q51V/Y52V-R	ACGAATAGTTTAGAAAAGGAATTACTC
A53V/K54V/Q55V-F	GCTGCTGCTTTAGACGTCAATATTGAA
A53V/K54V/Q55V-R	ATATTGTGTTATGTTCTTCTTGAAGTT
L56V/D57V/V58L-F	GCTGCTTGTGCTAATATTGAAAAGGTTT
L56V/D57V/V58L-R	CTTCGCATATTGTGTTATGTTCTTCTTG
N59V/I60V/E61V-F	GCTGCTGCTAAGGTTTATAAGGATAAAG
N59V/I60V/E61V-R	GACGTCTAATTGCTTCGCATATTGTGTT
K62V/V63L/Y64V-F	GCTGCTGCTAAGGATAAAGATAAAGG
K62V/V63L/Y64V-R	TTCAATATTGACGTCTAATTGCTTCG
K65V/D66V/K67V-F	GCTGCTGCTGATAAAGGTTCAGTCAAC
K65V/D66V/K67V-R	ATAAACCTTTTCAATATTGACGTCTAA
D68V/K69V/G70V-F	GCTGCTGCTTCAGTCAACGCTCAAAAGG
D68V/K69V/G70V-R	TTTATCCTTATAAACCTTTTCAATATTG
K62V-F	GCTGTTTATAAGGATAAAGATAAAGGT
K62V-R	TTCAATATTGACGTCTAATTGCTTCGC
V63L-F	TTGTATAAGGATAAAGATAAAGGTTCA
V63L-R	CTTTTCAATATTGACGTCTAATTGCTT
Y64V-F	GCTAAGGATAAAGATAAAGGTTCAGTC
Y64V-R	AACCTTTTCAATATTGACGTCTAATTG
K65V-F	GCTGATAAAGATAAAGGTTCAGTCAAC
K65V-R	ATAAACCTTTTCAATATTGACGTCTAA
D66V-F	GCTAAAGATAAAGGTTCAGTCAACGCT
D66V-R	CTTATAAACCTTTTCAATATTGACGTC
K67V-F	GCTGATAAAGGTTCAGTCAACGCTCAA
K67V-R	ATCCTTATAAACCTTTTCAATATTGAC
D68V-F	GCTAAAGGTTCAGTCAACGCTCAAAAG
D68V-R	TTTATCCTTATAAACCTTTTCAATATT
K69V-F	GCTGGTTCAGTCAACGCTCAAAAGGAT
K69V-R	ATCTTTATCCTTATAAACCTTTTCAAT
G70V-F	GCTTCAGTCAACGCTCAAAAGGATATC
G70V-R	TTTATCTTTATCCTTATAAACCTTTTC
**Primers used for site-directed mutagenesis of YycG_ex_ in the**	
***S. epidermidis* chromosome**	
^c^*yycG*-attB1	GGGGACAAGTTTGTACAAAAAAGCAGGCT
	ATGAAGTGGCTTAAACAACTAC
^c^*yycG*-attB2	GGGGACCACTTTGTACAAGAAAGCTGGGT
	ACAAGAAGGAATCACTATTTTCT

*^*a*^Primers were designed according to the genomic sequence of *S. epidermidis* RP62A (GenBank accession number NC_002976). F, forward primer; R, reverse primer. ^*b*^Underlined sequences represent *Nco*I and *Xho*I restriction sites. ^*c*^Underlined sequences represent the Gateway BP reaction sites.*

### Bacterial Dynamic Growth Curve

Overnight cultures of *S. epidermidis* RP62A were subcultured (1:200) in fresh TSB supplemented with 160 μg/mL YycG_ex_ mAbs and incubated at 37°C for 12 h with shaking. The effect of mAbs on bacterial growth was automatically monitored every 0.5 h by measuring the optical density at 600 nm (OD_600_) by using a BioScreen Growth Curve machine (Growth Curves USA, Piscataway, NJ, United States); normal mouse IgG (mIgG) served as the negative control. The experiments were performed in triplicate and the means and standard error of the mean (SEM) were calculated.

### Determination of Viable Bacterial Cell Counts

The viable cell counts of bacteria incubated with YycG_ex_ mAbs were determined through plate counts on nutrient agar. Overnight cultures of *S. epidermidis* RP62A were diluted 1:200 into TSB containing 160 μg/mL YycG_ex_ mAbs and incubated at 37°C with shaking for 6 or 24 h; subsequently, 1-mL aliquots of bacterial cells were washed twice with ice-cold 0.9% NaCl solution and pelleted by centrifugation for 3 min at 4000 rpm. The pellets were resuspended in 1 mL of ice-cold 0.9% NaCl solution and serially diluted (10-fold), and then 5-μL aliquots from each dilution were spotted onto tryptic soy agar plates and the CFU/mL values were calculated. Three independent experiments were performed for this assay.

### Microtiter Plate Biofilm Assays

Inhibition of biofilm formation by YycG_ex_ mAbs was detected using a semiquantitative microtiter plate assay. Overnight planktonic cultures of *S. epidermidis* RP62A were diluted 1:200 in fresh TSB containing twofold serial dilutions of mAbs (20, 40, 80, and 160 μg/mL), and then microtiter plates containing the samples were initially incubated at 4°C for 2 h and later placed in a 37°C incubator for 24 h. After the incubation and the removal of non-adherent cells by washing thrice with phosphate-buffered saline (PBS), adherent biofilms were fixed with 95% methanol and stained with 2% (w/v) crystal violet, and then excess stain was removed by washing in distilled water. To quantify biofilm formation, OD_570_ was determined using an xMark^TM^ Microplate Absorbance Spectrophotometer (Bio-Rad Laboratories, Hercules, CA, United States). Bacteria treated with mIgG served as the control. The experiments were performed using three replicates and repeated thrice independently.

### Structural Analysis of Biofilms Through Confocal Laser-Scanning Microscopy (CLSM)

Overnight cultures of *S. epidermidis* RP62A (1:200 dilution) were inoculated into 1 mL of TSB containing 80 μg/mL YycG_ex_ mAbs in FluoroDishes (FD35-100; WPI, Sarasota, FL, United States) and incubated statically at 37°C for 24 h. Planktonic cells were removed by washing with 0.9% NaCl solution, and biofilms were stained using a Live/Dead BacLight Viability kit (Thermo Fisher Scientific, Waltham, MA, United States) for 20 min at room temperature, protected from light exposure. We obtained a series of Z-section images by using a Leica confocal microscope (TCS-SP5, Leica, Wetzler, Germany) with a 63 × oil-immersion objective and then generated three-dimensional images by using IMARIS 7.0.0 software package (Bitplane, Zurich, Switzerland). Three independent experiments were performed.

### Bacterial Initial-Attachment Assays

Overnight-grown *S. epidermidis* RP62A cells were diluted 1:200 in TSB containing 160 μg/mL YycG_ex_ mAbs and incubated at 37°C with shaking at 220 rpm for 6 h. The bacteria were harvested by centrifugation and resuspended in PBS to an OD_600_ of 0.1 or 1.0 and then loaded in triplicate into either 6-well or 96-well polystyrene microtiter plates, respectively (Nunc GmbH, Wiesbaden, Germany). After incubation for 2 h at 37°C, the plates were washed thrice with PBS to remove non-adherent bacteria, and the adherent bacteria were stained with 0.5% crystal violet as described previously (Lei et al., 2011) and visualized microscopically using an Axiovert 200 inverted microscope (Carl Zeiss, Thornwood, NY, United States) or stained with 2% crystal violet and used for OD_570_ measurement. The experiments were performed using three replicates and repeated thrice independently.

### Detection of Polysaccharide Intercellular Adhesin (PIA)

Polysaccharide intercellular adhesin was detected by performing a lectin dot-blot assay with horseradish peroxidase (HRP)-conjugated wheat germ agglutinin (WGA-HRP, Biotium, Inc. Hayward, CA, United States) as previously described (Wu et al., 2015). Briefly, overnight cultures of *S. epidermidis* RP62A were diluted 1:200 in TSB containing 160 μg/mL YycG_ex_ mAbs and incubated at 37°C with shaking at 220 rpm for 6 h. The bacteria were harvested and resuspended in 50 μL of 0.5 M EDTA (pH 8.0) and then incubated for 5 min at 100°C. After centrifugation (13,000 × *g*, 5 min), the supernatant was treated with proteinase K (20 mg/mL; Roche) at 37°C for 2 h, after which the enzyme was inactivated at 100°C for 10 min. Next, 1-μL aliquots of serially diluted PIA extracts were spotted onto nitrocellulose membranes (Millipore) by using a 96-well dot-blot vacuum manifold (Biometra GmbH, Niedersachsen, Germany), and after air-drying, the membranes were blocked for 2 h with 3% (w/v) skim milk in Tris-buffered saline containing 0.1% Tween-20 (TBST). Subsequently, the membranes were incubated with WGA-HRP (2 μg/mL) for 1 h at room temperature, and after washing thrice with TBST, the PIA signal was visualized using an enhanced chemiluminescence (ECL) Western blotting detection kit (Thermo Fisher Scientific, Waltham, MA, United States).

### Detection of Extracellular DNA (eDNA)

Overnight cultures of *S. epidermidis* RP62A were diluted 1:200 into fresh TSB containing 160 μg/mL YycG_ex_ mAbs and incubated at 37°C with shaking for 24 h. Bacterial cells were harvested by centrifugation at 13,000 rpm for 10 min, and the resulting supernatants were filtered through 0.22-μm-pore-size filters (Millipore, Bedford, MA, United States) to obtain cell-free supernatants. The eDNA from the supernatants was isolated using an equal volume of phenol-chloroform-isoamyl alcohol (25:24:1, v/v/v) and then ethanol-precipitated. The precipitated eDNA was washed in 70% ethanol, air-dried, resuspended in TE buffer (10 mM Tris-Cl, 1 mM EDTA, pH 8.0), and visualized on gel-red-stained 1% agarose gels.

### Bacterial Autolysis Assay

To measure autolysis, overnight-grown *S. epidermidis* RP62A was diluted 1:200 into TSB containing 1 M NaCl plus 160 μg/mL YycG_ex_ mAbs and incubated at 37°C for 6 h. Next, the bacterial cells were pelleted by centrifugation, and the pellets were washed thrice with ice-cold water and resuspended to an OD_600_ of 1.0 in 50 mM glycine buffer, pH 8.0, containing 0.05% Triton X-100. Lastly, the bacterial cells were incubated at 37°C with shaking and the OD_600_ was measured at 0.5-h intervals for 3 h. All experiments were repeated thrice.

### RNA Isolation and qRT-PCR

Overnight cultures of *S. epidermidis* RP62A were diluted 1:200 into TSB containing 160 μg/mL YycG_ex_ mAbs and incubated at 37°C with shaking at 220 rpm for 6 h. The bacteria were collected by centrifugation at 4000 rpm for 10 min and then washed twice with ice-cold 0.9% NaCl solution. The cell pellets were homogenized by using 0.1-mm zirconia-silica beads in a Mini-BeadBeater (Biospec Products, Bartlesville, OK, United States), operated at a speed of 4800 rpm for 60 s; 5 cycles of homogenization were performed with 1-min intervals on ice. Total RNA was extracted using a RNeasy kit (Qiagen, Los Angeles, CA, United States) according to the manufacturer’s instruction. After RNA extraction, residual DNA was removed from the total RNA samples, and cDNA was synthesized by using a PrimeScript RT Reagent Kit with gDNA Eraser (Perfect Real Time) (Takara Bio Inc., Otsu, Japan) for manual description. qRT-PCR was performed on an Applied Biosystems 7500 Real Time PCR system (Applied Biosystems, Foster City, CA, United States) by using SYBR Premix Ex Taq (Takara Bio Inc., Otsu, Japan) and these cycling conditions: 95°C for 30 s, followed by 40 cycles of 95°C for 5 s and 60°C for 34 s. Melting curves were recorded after 40 cycles to confirm primer specificity by heating from 60°C to 95°C. All sample reactions were performed in triplicate with housekeeping gyrase B subunit gene (*gyrB*) used as an internal standard. Real-time PCR primers for each tested gene are listed in Table 1.

### Epitope Mapping by Using Recombinant Truncated YycG_ex_ Proteins

To locate the epitopes recognized by YycG_ex_ mAbs, YycG_ex_ protein was truncated using the following method. DNA fragments encoding truncated YycG_ex_ proteins were subcloned into the *Bam*HI and *Xho*I sites of a pET SUMO vector, and nine plasmids containing inserts of the desired length were verified through sequencing. All primers used in this study are listed in Table 1. *E. coli* strain BL21(DE3) carrying the aforementioned recombinant plasmids were incubated in LB medium at 37°C with shaking (at 220 rpm) until an OD_600_ of 0.6, and then 0.8 mM IPTG was added to induce expression at 25°C for 14 h; subsequently, cells were harvested by centrifugation and boiled in 400 μL of 1 × SDS sample buffer and stored at −20°C.

### Identification of Key Residues Within the Epitope by Using Site-Directed Mutagenesis

A KOD -Plus- Mutagenesis Kit (Toyobo, Osaka, Japan) was used to introduce alanine substitutions into the YycG_ex_ domain according to the manufacturer’s instructions. The plasmid pET SUMO expressing the truncated YycG_ex_ fragment TF_34__–__83_ was used as a template. The amplification conditions included an initial denaturation step of 2 min at 94°C, followed by 10 cycles of 10 s at 98°C, 30 s at 55°C, and 6 min at 68°C. The mutant plasmids were verified using DNA sequencing before use in transformation of *E. coli* strain BL21(DE3). The mutagenesis primers used in the assay are listed in Table 1.

### Analysis of Binding of Truncated YycG_ex_ Proteins and YycG_ex_ mAbs by Using SDS-PAGE and Western Blotting

Cell pellets obtained from 5 mL of LB medium were boiled in 400 μL of 1 × SDS sample buffer for 10 min, and total proteins were separated using 10% (w/v) SDS-PAGE. Gels were either stained with Coomassie Brilliant Blue R-250 or the proteins were electrophoretically transferred onto 0.45-μm PVDF membranes (Millipore, Bedford, MA, United States). After blocking for 2 h with 5% skim milk in PBST (PBS containing 0.05% Tween-20), the membranes were incubated with YycG_ex_ mAbs (1:1000 in PBST) at room temperature for 3 h. Binding was probed using HRP-conjugated anti-mouse IgG, and after washing for 60 min with PBST, band intensity was measured by staining with an ECL detection reagent (Thermo Fisher Scientific, Waltham, MA, United States), according to the manufacturer’s instructions, and exposing the membranes to X-OMAT film (Kodak, Rochester, NY, United States).

### Construction of YycG Mutated in Key Residue Involved in Binding YycG_ex_ mAbs

To identify the function of a key amino acid residue recognized by YycG_ex_ mAbs in *S. epidermidis* RP62A, we next constructed a *yycG* (K65V) mutant in the bacterial chromosome by means of allelic replacement, performed using the temperature-sensitive plasmid pKOR1 as described previously (Bae and Schneewind, 2006). Briefly, a 1.0-kb DNA fragment (1∼1000 bp of *yycG*) was PCR-amplified from *S. epidermidis* RP62A genomic DNA (GenBank accession number NC_002976) by using the primers yycG-attB1/2 and then cloned into pKOR1 by using the Gateway BP cloning method, which yielded the plasmid pKOR1-*yycG*_1000_. We next used primers K65V-F (5′-GCTGATAAAGATAAAGGTTCAGTCAAC-3′) and K65V-R (5′-ATAAACCTTTTCAATATTGACGTCTAA-3′) harboring the K65V mutation together with the plasmid pKOR1-yycG_1000_ as the template and used a KOD -Plus- Mutagenesis Kit (Toyobo, Osaka, Japan) according to the manufacturer’s instructions. The mutated plasmid was first transformed into *E. coli* DC10B and then into *S. epidermidis* RP62A, and this was followed by allelic replacement as described previously (Zhu et al., 2010; Lou et al., 2011). The presence of the mutation (K65V) in the *S. epidermidis* genome was verified by sequencing the PCR product of YycG_ex_ amplified from the mutant genome.

### Statistical Analysis

Experiments were performed using three replicates and repeated thrice independently. Results are expressed as means ± standard deviation (SD) or SEM. For statistical comparisons, Student’s *t*-tests were performed using GraphPad Prism version 7 (GraphPad Software Inc., San Diego, CA, United States). *P* < 0.05 was considered statistically significant.

## Results

### Effect of YycG_ex_ mAbs on the Phenotypic Properties of *S. epidermidis* RP62A

YycG/YycF TCS regulates *S. epidermidis* biofilm formation (Xu et al., 2017). Because inhibitors targeting the cytoplasmic HATPase_c domain of YycG exhibit anti-biofilm activity (Qin et al., 2006; Huang et al., 2012; Liu et al., 2014; Lv et al., 2017), we generated mAbs against the extracellular domain of YycG (YycG_ex_, aa 34–184) of *S. epidermidis* RP62A, and from nine hybridoma lines, we selected for further investigation the two mAbs (2F3 and 1H1) that exhibited the highest affinity for the protein.

#### Effect of YycG_ex_ mAbs on Biofilm Formation

**FIGURE 1:**
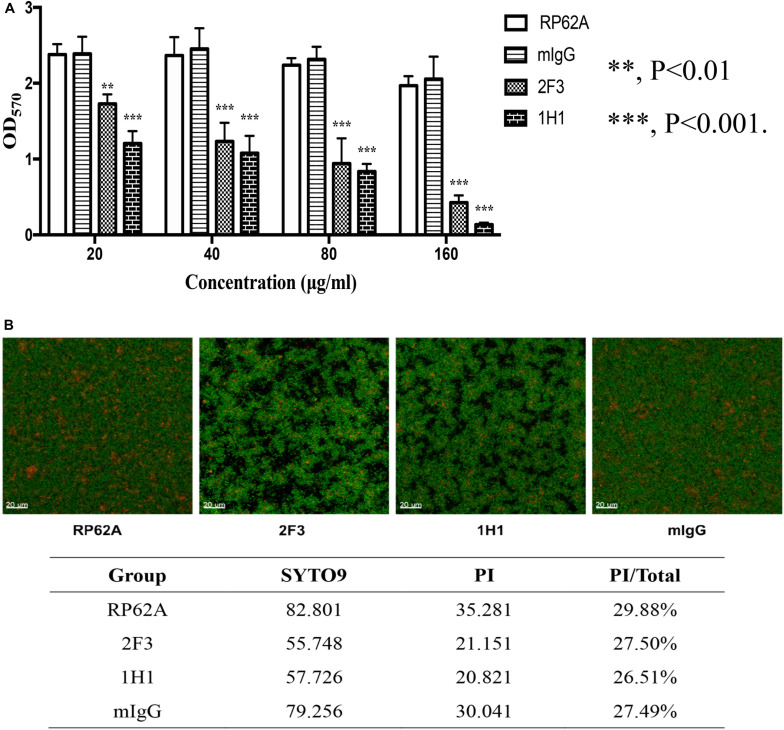
Effects of YycG_ex_ mAbs on the biofilm formation of *S. epidermidis* RP62A. **(A)** Inhibition of biofilm formation by YycG_ex_ mAbs was detected using a microtiter plate assay. *S. epidermidis* RP62A was incubated with twofold serial dilutions of YycG_ex_ mAbs (160–20 μg/mL) at 37°C for 24 h. Biofilms were stained with 2% (w/v) crystal violet and the OD_570_ was read. Statistical analyses were performed using Student’s *t*-test. **(B)** Effect of YycG_ex_ mAbs on biofilm morphology of *S. epidermidis* RP62A, examined using CLSM. *S. epidermidis* RP62A was incubated with 80 μg/mL YycG_ex_ mAbs at 37°C for 24 h. Viable and dead cells fluoresced green and red, respectively. PI/Total values indicated the proportion of dead cells within the biofilms. RP62A, untreated; mIgG, normal mouse IgG treated.

To test the effect of YycG_ex_ mAbs on the biofilm formation of *S. epidermidis* RP62A, 20–160 μg/mL YycG_ex_ mAbs were incubated with the bacteria for 24 h at 37°C, and then biofilm formation was evaluated using a microtiter plate assay; here, mIgG-treated and untreated groups were used as controls. YycG_ex_ mAbs inhibited *S. epidermidis* biofilm formation in a dose-dependent manner (Figure 1A), and mAbs 2F3 and 1H1 at 20 μg/mL reduced biofilm formation by 27.3 and 49.2%, respectively, relative to the control groups. At 160 μg/mL, mAbs 2F3 and 1H1 caused 78.3 and 93.1% biofilm reduction, respectively, whereas the mIgG-treated group only showed 5.9% inhibition. After incubation with YycG_ex_ mAbs (80 μg/mL) for 24 h at 37°C, *S. epidermidis* RP62A formed rough and loose biofilms, as observed by means of Live/Dead staining and CLSM, whereas the biofilms in the mIgG-treated and untreated groups were smooth and evenly distributed. However, the proportions of dead bacteria in the biofilm were similar in all groups (Figure 1B). YycG_ex_ mAbs 2F3 and 1H1 did not affect the growth and survival of *S. epidermidis* RP62A at the highest concentration tested (160 μg/mL) (Supplementary Figure 1).

#### YycG_ex_ mAbs Influence Bacterial Initial Attachment

**FIGURE 2:**
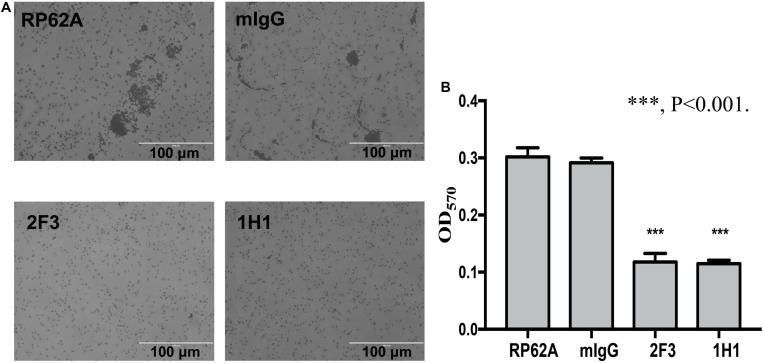
Effect of YycG_ex_ mAbs on initial attachment of *S. epidermidis* RP62A. Bacterial cells were diluted in TSB containing 160 μg/mL YycG_ex_ mAbs and incubated at 37°C with shaking at 220 rpm for 6 h. **(A)** Adherence ability visualized microscopically. **(B)** Cell attachment measured as OD_570_ after crystal violet staining. Experiments were performed using three replicates and repeated thrice independently. RP62A: untreated; mIgG: normal mouse IgG treated. Statistical analyses were performed using Student’s *t*-test. ^∗∗∗^*P* < 0.001.

The first step in biofilm formation is bacterial adherence to a surface; thus, we determined how this initial attachment of *S. epidermidis* is affected by YycG_ex_ mAbs, and we used a semiquantitative microtiter plate assay to assess cell attachment. Under the microscope, no cell clusters were detected when *S. epidermidis* RP62A was cultured in the presence of YycG_ex_ mAbs for 6 h at 37°C, whereas clusters were observed in the mIgG-treated and untreated groups (Figure 2A). The adherent bacteria were quantitated by staining with crystal violet and measuring the OD_570_: Both mAb 2F3 and mAb 1H1 significantly reduced the number of attached cells in the wells (OD_570_ = 0.118 ± 0.012 and 0.115 ± 0.005, respectively) as compared to that in the untreated control group (OD_570_ = 0.302 ± 0.013) (*P* < 0.001) (Figure 2B).

### Effect of YycG_ex_ mAbs on Extracellular Polysaccharide Substance (EPS)

**FIGURE 3:**
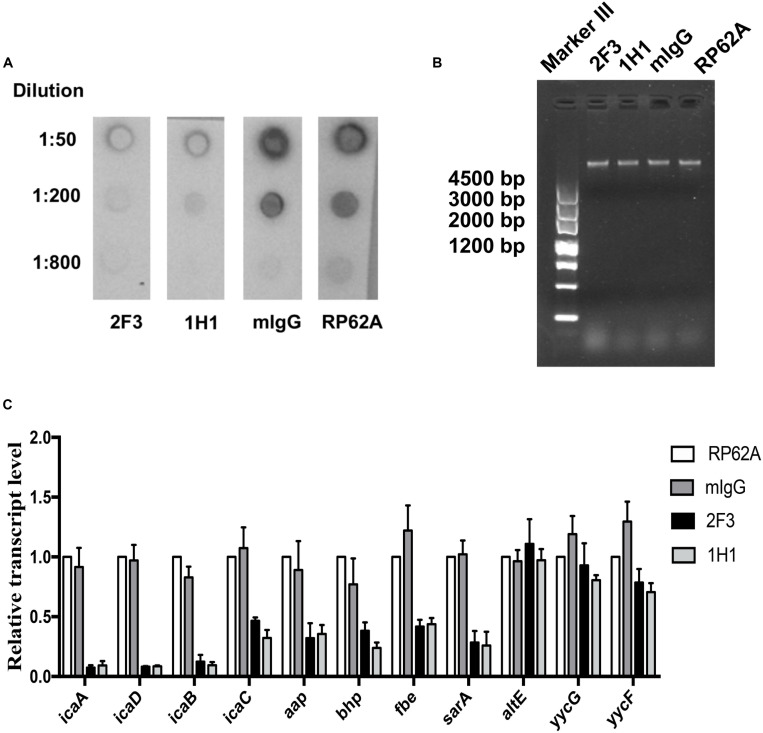
Effect of YycG_ex_ mAbs on EPS synthesis and transcription of biofilm-related genes of *S. epidermidis* RP62A. Overnight cultures of *S. epidermidis* RP62A were diluted in TSB containing 160 μg/mL YycG_ex_ mAbs and incubated at 37°C for 6 h. **(A)** Effect of YycG_ex_ mAbs on PIA synthesis. **(B)** Effect of YycG_ex_ mAbs on eDNA release. eDNA was isolated and analyzed on 1% agarose gels. **(C)** Effect of YycG_ex_ mAbs on transcriptional levels of biofilm-related genes. All reactions were performed in triplicate, with housekeeping gyrase B subunit gene (*gyrB*) used as an internal standard. RP62A, untreated; mIgG, normal mouse IgG treated.

To investigate the effect of YycG_ex_ mAbs on EPS production, PIA biosynthesis and eDNA release were determined in the case of *S. epidermidis* RP62A cultured with 160 μg/mL YycG_ex_ mAbs at 37°C for 6 h. Almost no PIA was detected in the presence of 160 μg/mL YycG_ex_ mAbs at dilutions up to 1:50 by using WGA-HRP in a lectin dot-blot assay, whereas a considerably stronger signal was detected in the mIgG-treated and untreated groups at dilutions up to 1:200 (Figure 3A). The release of eDNA into the supernatant of cells treated with YycG_ex_ mAbs was similar to that in the untreated *S. epidermidis* RP62A cells, as visualized on gel-red-stained 1% agarose gels (Figure 3B).

We next assessed the transcriptional levels of the biofilm-related genes *icaADBC*, *aap*, *bhp*, *fbe*, and *sarA* by performing qRT-PCR with RNA samples extracted from *S. epidermidis* RP62A incubated with 160 μg/mL mAbs 2F3 and 1H1 at 37°C for 6 h. All of these genes were downregulated in the presence of YycG_ex_ mAbs, with a marked (8–13-fold) reduction in transcript levels being measured for *icaA*, *icaD*, and *icaB*, which encode PIA; by contrast, the expression of *altE, yycG*, and *yycF* did not differ between the mAb-treated and untreated groups (Figure 3C).

### Epitope Mapping and Identification of Key Amino Acid Residues in the YycG_ex_ Domain

Localization of the B-cell epitopes recognized by YycG_ex_-specific mAbs can help in identifying the main antigenic determinants of the YycG_ex_ domain and developing peptide vaccines for *S. epidermidis* biofilm infections. Thus, we mapped the epitopes within YycG_ex_ domain by using protein truncation combined with Western blotting analysis. Moreover, by performing alanine-scanning mutagenesis of YycG_ex_ proteins, we identified multiple residues that are essential for YycG_ex_ mAb binding.

#### Identification of Epitopes Recognized by YycG_ex_ mAbs

**FIGURE 4:**
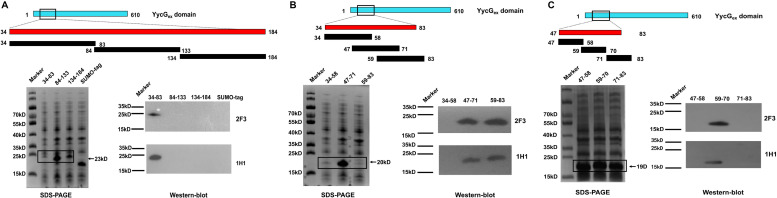
Epitope mapping of YycG_ex_ mAbs within YycG_ex_ domain. YycG_ex_ domain was truncated into fragments of distinct sizes harboring an N-terminally fused SUMO-tag, as shown in the schematic diagrams; the specific recognition of the truncation fragments (TFs) by YycG_ex_ mAbs was assessed through Western blotting. Binding of YycG_ex_ mAbs to **(A)** TF_34__–__83_, TF_84__–__133_, and TF_134__–__184_; **(B)** TF_34__–__58_, TF_47__–__71_, and TF_59__–__83_; and **(C)** TF_47__–__58_, TF_59__–__70_, and TF_71__–__83_.

To locate the epitopes recognized by YycG_ex_ mAbs, we generated YycG_ex_ truncation fragments (TFs) of distinct sizes that were N-terminally fused to a SUMO-tag, and we used Western blotting to examine the binding of YycG_ex_ mAbs to these fragments. First, YycG_ex_ domain was truncated into three fragments: TF_34__–__83_, TF_84__–__133_, and TF_134__–__184_. Both mAb 2F3 and mAb 1H1 recognized TF_34__–__83_ (Figure 4A). Next, TF_34__–__83_ was further truncated into TF_34__–__58_, TF_47__–__71_, and TF_59__–__83_; mAbs 2F3 and 1H1 both reacted with TF_47__–__71_ and TF_59__–__83_, which indicated that the YycG_ex_ mAbs recognize the region containing aa 47–83 (Figure 4B). Lastly, the fragment containing aa 47–83 of the YycG_ex_ domain was truncated into TF_47__–__58_, TF_59__–__70_, and TF_71__–__83_, and YycG_ex_ mAbs were found to bind TF_59__–__70_ (Figure 4C).

#### Identification of Key Amino Acid Residues Within the mAb Epitope

**FIGURE 5:**
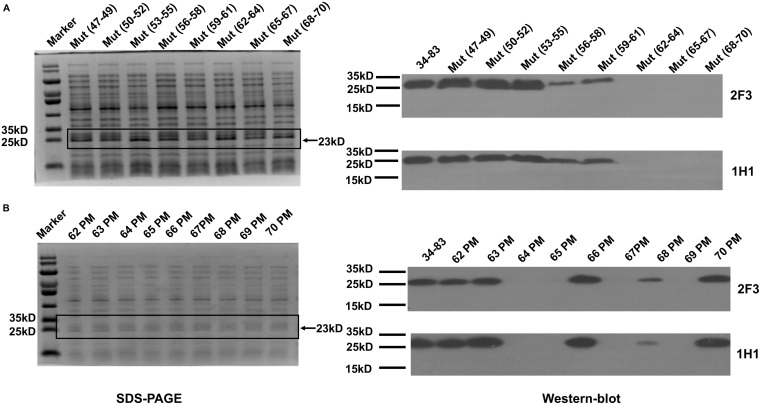
Identification of critical amino acid residues in YycG_ex_ domain epitope. Specific recognition by YycG_ex_ mAbs of TF_34__–__83_ harboring alanine substitutions was assessed using Western blotting. Binding of YycG_ex_ mAbs to TF_34__–__83_ carrying **(A)** triple-alanine substitutions and **(B)** single-alanine substitutions.

To identify the amino acid residues in the epitope that are essential for binding by YycG_ex_ mAbs, we substituted every three amino acid residues with alanine individually within aa 47–70 by using site-directed mutagenesis; we constructed eight mutants and used Western blotting to examine their binding by YycG_ex_ mAbs. Binding of the epitope by both mAb 2F3 and mAb 1H1 was abolished by the mutations K62V/V63L/Y64V, K65V/D66V/K67V, and D68V/K69V/G70V, which indicated that the key amino acid residues in the epitope recognized by YycG_ex_ mAbs are located within aa 62–70: KVYKDKDKG (Figure 5A). Consequently, we mutated these nine amino acid residues individually to alanine and then examined antibody binding; the binding of mAbs 2F3 and 1H1 to the epitope was abolished by four single mutations, Y64V, K65V, K67V, and K69V, which suggested that the key amino acid residues in the YycG_ex_ mAb epitope were Tyr64, Lys65, Lys67, and Lys69 (Figure 5B).

#### YycG K65V Mutation in *S. epidermidis* Decreases Biofilm Inhibition by YycG_ex_ mAbs

Lastly, to evaluate how the key amino acids in the mAb epitope (Tyr64, Lys65, Lys67, and Lys69) affect biofilm inhibition by YycG_ex_ mAbs, we generated *yycG* mutations in *S. epidermidis* RP62A (Y64V/K65V/K67V/K69V, Y64V, and K65V) through allelic replacement by using the temperature-sensitive plasmid pKOR1, but only the *yycG* (K65V)-mutant strain was screened out; viable mutant strains were not obtained in the case of the other mutations mentioned. The *yycG* (K65V) mutant was verified by sequencing the PCR product of YycG_ex_ amplified from the mutant genome, and pKOR1 plasmid loss from mutant strains was confirmed through PCR amplification and agarose-gel analysis (Supplementary Figure 2A).

**FIGURE 6:**
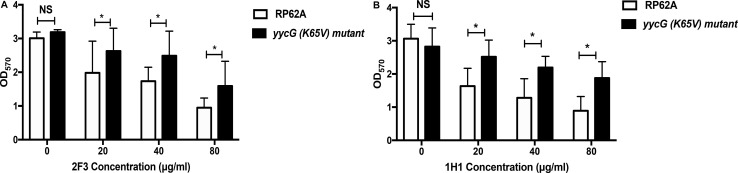
Effect of YycG_ex_ mAbs on biofilm formation of the *yycG* (K65V) mutant. Inhibition of biofilm formation by YycG_ex_ mAbs was detected using a semiquantitative plate assay. Overnight cultures were diluted in fresh TSB containing twofold serial dilutions of YycG_ex_ mAbs (80–20 μg/mL) and incubated at 37°C for 24 h. Biofilms were stained with 2% crystal violet, and OD_570_ was measured. Effect of mAb 2F3 **(A)** and mAb 1H1 **(B)** on the biofilm formation of the *yycG* (K65V) mutant and *S. epidermidis* RP62A. Experiments were performed using three replicates and repeated thrice independently. Statistical analysis was performed using Student’s *t*-test. *P < 0.05; NS, not significant.

Growth curves and biofilm formation were no different between the *yycG* (K65V) mutant strain and the parental strain (Supplementary Figures 2B,C). However, the inhibitory effect of YycG_ex_ mAbs on biofilm formation of the *yycG* (K65V) mutant was significantly decreased with increasing concentrations of the antibodies (20, 40, and 80 μg/mL) as compared with the inhibition in the parental RP62A strain (*P* < 0.05) (Figure 6). At 80 μg/mL, mAbs 2F3 and 1H1 reduced the biofilm formation of RP62A strain by 68.5 and 70.9%, respectively, but caused only 50.0 and 33.5% reduction in the *yycG* (K65V) mutant.

## Discussion

*Staphylococcus epidermidis* has emerged as one of the most critical opportunistic pathogens of nosocomial infections in recent years because of the increase in frequency of invasive procedures (Vuong et al., 2003; Chessa et al., 2015). The main pathogenicity associated with *S. epidermidis* involves the formation of biofilms on implanted medical devices (Rupp, 2014; Hamad et al., 2015). The presence of the bacteria in biofilms drastically reduces the efficacy of conventional antibiotics and the host immune system, and this leads to persistent or recurrent infections (Wolcott et al., 2010; Romling and Balsalobre, 2012). The YycG/YycF TCS is essential for bacterial growth and plays a major role in biofilm formation (Fukuchi et al., 2000; Howell et al., 2003; Mohedano et al., 2005; Ahn and Burne, 2007; Bisicchia et al., 2007). YycG histidine kinase is a transmembrane protein, and we have shown that inhibitors that target the cytoplasmic HATPase_c domain of *S. epidermidis* exhibit bactericidal and biofilm-killing activities (Qin et al., 2006; Huang et al., 2012; Liu et al., 2014; Lv et al., 2017). Previous studies have found that the extracellular domain of the protein (YycG_ex_ domain) adopts a PAS fold predicted as a sensor of YycG by sensing environmental signals such as oxygen, light, redox potential, or the presence of specific ligands (Taylor and Zhulin, 1999; Santelli et al., 2007; Chang et al., 2010; Shah et al., 2013; Kim et al., 2016), but little is known of the nature of the signals and factors sensed by YycG. However, whether the blockade of YycG_ex_ domain would influence the signal transduction and biofilm formation of the bacterium has remained unclear. Therefore, in this study, we investigated the effect of mAbs against the YycG_ex_ domain on the biological functions of *S. epidermidis* RP62A.

We selected two mAbs showing the highest affinity for YycG_ex_ protein, mAbs 2F3 and 1H1, and we found that both mAbs reduced the biofilm formation of *S. epidermidis* RP62A in a dose-dependent manner under static conditions without affecting bacterial growth. Furthermore, both mAb 2F3 and mAb 1H1 reduced the biofilm formation of five *S. epidermidis* clinical isolates by 54.1–74.7% at the concentration of 160 μg/mL (Supplementary Table 1), which suggests that YycG_ex_ mAbs represent potential candidates for use in clinical treatments to prevent biofilm formation. However, the cell wall of *S. epidermidis* is formed by a thick layer (20–80 nm) composed mainly of glycine-rich peptidoglycan that might impede the binding of YycG_ex_ mAbs to the YycG_ex_ domain, and this could be responsible for the high concentration of YycG_ex_ mAbs (160 μg/mL) required for producing their effect. Thus, further investigation is necessary to develop methods to reduce the treatment concentration of YycG_ex_ mAbs while retaining their strong biofilm-inhibition activity.

We found that YycG_ex_ mAbs diminished the capacity of *S. epidermidis* for initial attachment, which is the first step in biofilm formation. Biofilm formation is commonly divided into three steps: initial adhesion, accumulation, and maturation (Sauer, 2003; Wolska et al., 2016). After attaching to a surface, bacteria produce the EPS, a complex mixture of biopolymers primarily consisting of polysaccharides (PIA), proteins, and nucleic acids (eDNA) (Costerton et al., 1987; Vu et al., 2009). We detected the expression of genes (*aap*, *bhp, fbe*, and *sarA*) encoding proteins related to the initial attachment and accumulation of the *S. epidermidis* biofilm (Mack et al., 1994; Cho et al., 2002; Bowden et al., 2005; Vuong et al., 2005; Schaeffer et al., 2015), and all of these genes were downregulated after treatment with YycG_ex_ mAbs. The downregulation of *icaADB* expression was in accord with the decrease in PIA synthesis: The *ica* operon encodes PIA-synthesis enzymes, and PIA is a poly-β (1–6)-*N*-acetylglucosamine that plays critical roles in intercellular adhesion and in the structural integrity of biofilms (Heilmann et al., 1996; Gerke et al., 1998; Lister and Horswill, 2014; Arciola et al., 2015). Another key EPS component of biofilms is the eDNA released after bacterial autolysis, and this eDNA is related to initial cell adhesion and subsequent intracellular accumulation (Mann et al., 2009; Tang et al., 2013; Regina et al., 2014; Ibanez de Aldecoa et al., 2017). The release of eDNA into the supernatant and the Triton X-100-induced autolysis of the cells that were treated with YycG_ex_ mAbs were similar to those of untreated *S. epidermidis* RP62A cells (Supplementary Figure 3). The signals in the environment induce auto-phosphorylation of the cytoplasmic domain HATPase_c of YycG, and the phosphoryl group is then transferred from histidine residue to an aspartate residue in the receiver domain of the YycF response regulator, which further results in the conformation change of YycF (Mohedano et al., 2005; Cronan and Thomas, 2009). Phosphorylated YycF is usually involved in the DNA-binding and transcription activation of the target genes. The previous study in our lab showed that in *S. epidermidis*, YycF not only regulates biofilm-associated regulators such as *arlRS*, *sarA*, and *sarX* but also binds to the promoters of *icaADBC* to directly modulate PIA production, which related with bacterial biofilm formation (Xu et al., 2017). Thus, YycG_ex_ mAbs might inhibit biofilm formation by affecting the signal transduction from YycG to YycF, which led to the downregulated expression level of *icaADBC* and diminished initial-adherence capacity and, thus, likely influenced the subsequent accumulation step.

Next, we determined that the epitope recognized by YycG_ex_ mAbs is located within aa 59–70, and by analyzing the antibody-binding region harboring alanine substitutions, we identified four critical amino acids in the epitope: Tyr64, Lys65, Lys67, and Lys69. To further evaluate how these key amino acids affect biofilm inhibition by YycG_ex_ mAbs, we generated *yycG* mutations in the *S. epidermidis* chromosome (Y64V/K65V/K67V/K69V, Y64V, and K65V). Ultimately, we screened out the *yycG* (K65V) mutant, and we found that YycG_ex_ mAbs more weakly affected the biofilm formation of the *yycG* (K65V) mutant as compared with that of the parental RP62A strain. This finding indicated that the residue K65 might be directly involved in the binding of YycG_ex_ mAbs to YycG_ex_ domain. However, we failed to obtain the mutants *yycG* (Y64V/K65V/K67V/K69V) and *yycG* (Y64V) in *S. epidermidis* RP62A, which indicates that some of these residues likely play a crucial role in maintaining bacterial growth and survival.

In summary, YycG_ex_ mAbs showed anti-biofilm activity in the case of *S. epidermidis* and the epitope recognized by the mAbs was located in aa 59–70 of the YycG_ex_ domain. The key amino acids within the epitope were identified as Tyr64, Lys65, Lys67, and Lys69, and the importance of Lys65 was confirmed in *S. epidermidis* RP62A through single-point chromosome mutagenesis. Our findings suggest that mAbs against the YycG_ex_ domain could be used in new treatment strategies targeting biofilm-associated infections caused by S. *epidermidis*. Moreover, the epitope recognized by YycG_ex_ mAbs could be used to further study the structure of the YycG_ex_ domain and might represent a vaccine candidate for the prevention of *S. epidermidis* biofilm infections. However, further investigation is necessary to elucidate the mechanism by which YycG_ex_ mAbs block *S. epidermidis* signal transduction. Moreover, the effect of YycG_ex_ mAbs against biofilm-associated infections of *S. epidermidis* needs to be further investigated in a stable animal model.

## Data Availability Statement

The raw data supporting the conclusions of this article will be made available by the authors, without undue reservation.

## Ethics Statement

All mice experimental procedures were approved by the Institutional Animal Care and Use Committee (IACUC) of School of Basic Medical Sciences, Fudan University (IACUC Animal Project Number 20150119-081) according to Regulations for the Administration of Affairs Concerning Experimental Animals, China.

## Author Contributions

ZL and DQ conceived and designed the experiments. ZL, YS, XW, YW, JZ, HL, TG, LY, and DQ performed the experiments. ZL, YS, and DQ created the figures, analyzed the data, and wrote the manuscript. All the authors contributed to the article and approved the submitted version.

## Conflict of Interest

The authors declare that the research was conducted in the absence of any commercial or financial relationships that could be construed as a potential conflict of interest.

## Footnotes

1 http://www.ab-mart.com
