# Supplementary material for: Monoclonal Antibodies Specific to the Extracellular Domain of Histidine Kinase YycG of Staphylococcus epidermidis Inhibit Biofilm Formation
Source: Front Microbiol. 2020 Aug 7;11:1839. doi: 10.3389/fmicb.2020.01839 (PMC7426370; doi:10.3389/fmicb.2020.01839)
Supplement: Supplementary file 1 [file Data_Sheet_1.docx]

**Supplementary Material**

**
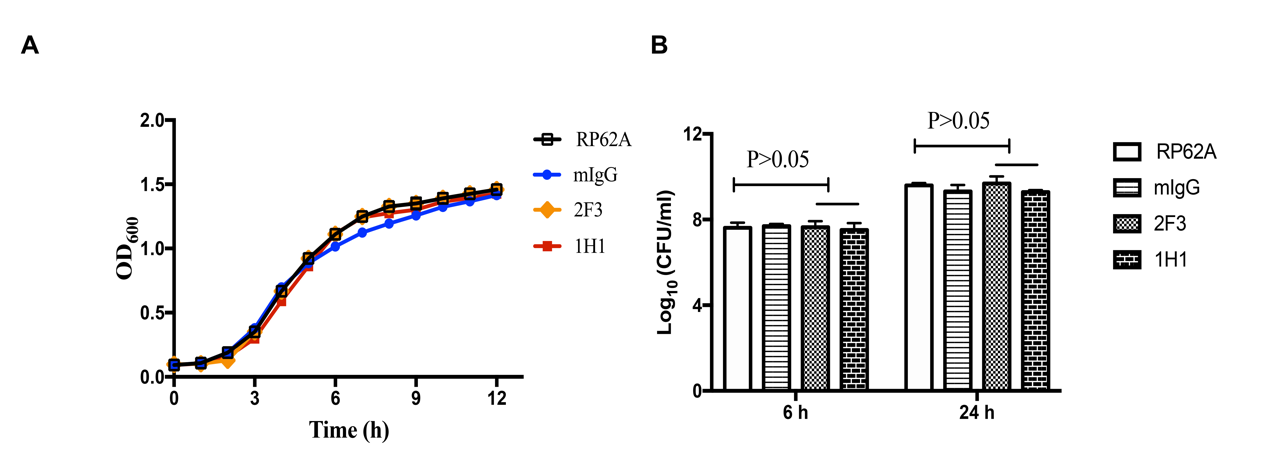
**

**Figure S1.** Impact of YycG_ex_ mAbs on growth and cell viability of *S. epidermidis* RP62A. **(A)** Growth curve of *S. epidermidis* RP62A incubated with 160 μg/mL YycG_ex_ mAbs. **(B)** Effect of YycG_ex_ mAbs (160 μg/mL) on bacterial viability. RP62A: untreated; mIgG: normal mouse IgG treated. Results shown are means and SD of triplicate samples. Data are the means of representative triplicates from three independent experiments. Statistical analyses were performed using Student’s *t* test.


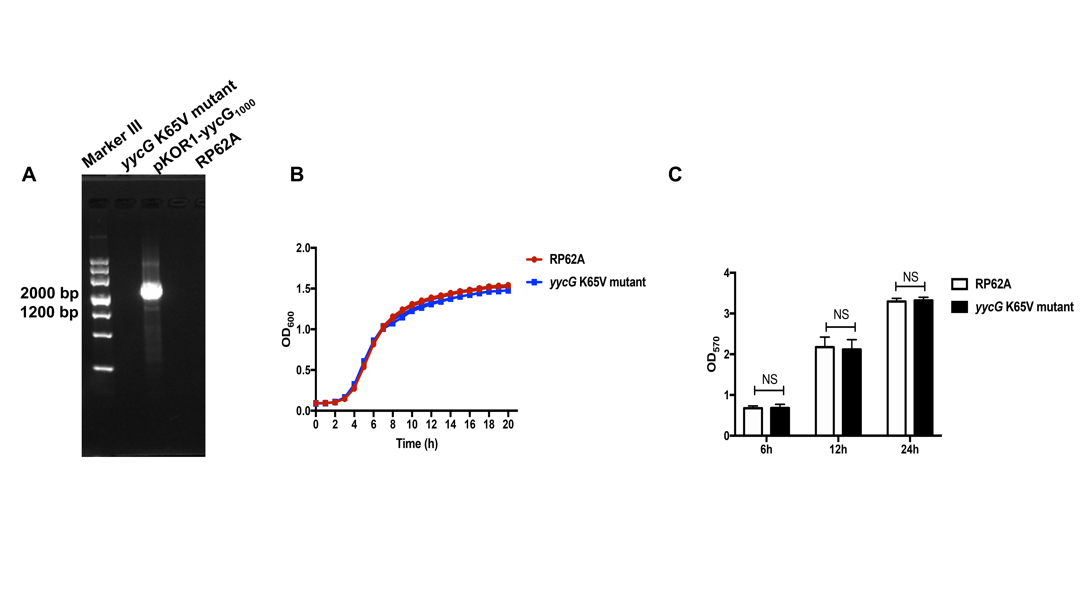


**Figure S2.** Growth curve and biofilm formation of *S. epidermidis* RP62A (65aa mut). **(A)** PCR amplification-based validation of pKOR1 plasmid loss from RP62A (65aa mut) strain genomic DNA. **(B)** Growth curve of *S. epidermidis* RP62A (65aa mut). Each data point represents three replicates and is shown together with the SEM. (C) Biofilm-forming capacity of *S. epidermidis* RP62A (65aa mut). Data shown are means of representative triplicates from three independent experiments. NS, not significant.

**Figure S3.** Effect of YycG_ex_ mAbs on autolysis of *S. epidermidis* RP62A. Triton X-100-induced autolysis of *S. epidermidis* RP62A in the presence of 160 μg/mL YycG_ex_ mAbs. Bacteria were incubated with YycG_ex_ mAbs at 37°C for 6 h, washed with ice-cold distilled water, and resuspended in 0.05% Triton X-100. OD_600_ was monitored at 30-min intervals. Data shown are means ± SD of three independent experiments. RP62A: untreated; mIgG: normal mouse IgG treated.

**Table S1.** Inhibition by YycG_ex_ mAbs of biofilm formation (24 h) of clinical staphylococcal strains.

| Clinical strains | | ^a^Inhibition rate (%) | |
| --- | --- | --- | --- |
|  |  | mAb 2F3 | mAb 1H1 |
|  | 1020081 | 65.6 | 59.8 |
|  | 1019975 | 68.8 | 68.7 |
|  | 1017872 | 74.6 | 66.2 |
|  | 1000153 | 71.2 | 58.2 |
|  | 1020235 | 54.1 | 70.0 |

^a^YycG_ex_ mAbs were used at the concentration of 160 μg/mL, and mAb inhibition of biofilm formation was estimated using a semiquantitative plate assay.
